# Mobile App for Parental Empowerment for Caregivers of Children With Autism Spectrum Disorders: Prospective Open Trial

**DOI:** 10.2196/27803

**Published:** 2021-09-15

**Authors:** Olivier Bonnot, Vladimir Adrien, Veronique Venelle, Dominique Bonneau, Fanny Gollier-Briant, Stephane Mouchabac

**Affiliations:** 1 Child and Adolescent Psychiatry Department Centre Hospitalier Universitaire de Nantes Nantes France; 2 Centre Ressource Autisme Angers France; 3 LPPL, EA 4638, University of Nantes Nantes France; 4 Department of Psychiatry Hôpital St Antoine Assistance Publique Hôpitaux de Paris Paris France; 5 Infrastructure of Clinical Research in Neurosciences Brain and Spine Institute Institut national de la santé et de la recherche médicale, Sorbonne University Paris France; 6 Department of Biochemistry and Genetics Centre Hospitalier Universitaire, Angers Angers France

**Keywords:** autism spectrum disorders, empowerment, smartphone application, autism, smartphone, app, children, caregivers

## Abstract

**Background:**

Conflicting data emerge from literature regarding the actual use of smartphone apps in medicine; some considered the introduction of smartphone apps in medicine to be a breakthrough, while others suggested that, in real-life, the use of smartphone apps in medicine is disappointingly low. Yet, digital tools become more present in medicine daily. To empower parents of a child with autism spectrum disorder, we developed the Smartautism smartphone app, which asks questions and provides feedback, using a screen with simple curves.

**Objective:**

The purpose of this study was to evaluate usage of the app by caregivers of individuals with autism spectrum disorders.

**Methods:**

We conducted a prospective longitudinal exploratory open study with families that have a child with autism spectrum disorder. Data were recorded over a period of 6 months, and the outcome criteria were (1) overall response rates for a feedback screen and qualitative questionnaires, and (2) response rates by degree of completion and by user interest, based on attrition.

**Results:**

Participants (n=65) had a very high intent to use the app during the 6-month period (3698/3900 instances, 94.8%); however, secondary analysis showed that only 46% of participants (30/65) had constant response rates over 50%. Interestingly, these users were characterized by higher use and satisfaction with the feedback screen when compared to low (*P*<.001) and moderate (*P*=.007) users.

**Conclusions:**

We found that real or perceived utility is an important incentive for parents who use empowerment smartphone apps.

**International Registered Report Identifier (IRRID):**

RR2-10.1136/bmjopen-2016-012135

## Introduction

Autism spectrum disorder is a chronic disorder that affects daily life and can be a burden on parents, with consequences on their quality of life [1,2]. Parents of children with autism spectrum disorders have greater anxiety levels than parents of children without developmental disorders [3]. Some major autism symptoms, such as communication disorders and aggression [4], along with behavioral symptoms (such as agitation, feeding difficulties, sleep disturbances, obsessive behavior, or refusal of authority), are well-known stressors.

Management of children’s inappropriate behaviors by professionals (or advice from professionals) is not accessible at all times of the day, and families often criticize the short duration or low frequency of their consultations [5-9]. Digital tools might be of particular interest in such situations by filling the gap between psychiatric consultations.

Digital mental health interventions have been of interest to the medical community for the past decade. Patients, caregivers, and medical practitioners are surrounded by apps and digital tools in everyday life; therefore, it seems natural that the mental health community, despite their initial criticism and poor initial acceptance [10], should now attempt to understand and develop digital mental health interventions programs. The benefits of digital mental health interventions are known: (1) low cost at a time when there are not enough professionals to help all patients, (2) no commute, and thus a gain of time, (3) increased patient involvement, and (4) increased patient accessibility, when needed. However, recent studies [11-13] have shown a gap between the alleged, and sometimes scientifically validated, purposes of such digital interventions and their actual use by patients and professionals. The American Psychiatric Association proposed a 5-level hierarchical framework to evaluate an app: quality of information, basic medical decision making (and nonmaleficence), scientific evidence usability, and interoperability [14].

We developed Smartautism—a smartphone app for parents with children with autism spectrum disorders—to meet the need for parental support between medical appointments. The app collects data through ecological momentary assessment [15] or the experience sampling method [16] from parents, who answer simple questions about daily life [17]. The app then provides a feedback screen with a graphical representation of their score, allowing parents to see the evolution of the scores during the weeks before to help parents be objective about the difficulties they are facing

But, even for tools with state-of-the-art development, validity, and usefulness, the rate of attrition may remain high. Eysenbach [18] defined this phenomenon as “nonusage attrition” and proposed influential factors such as the nature of the perceived advantage of an innovation. Likewise, usability, determined by its complexity, contributes to intention to use. It is fundamental that participants fit within the intended scope of users of the apps; therefore, appropriate information about the main purpose of the apps must be provided in order to avoid unrealistic user expectations. Finally, the nature of the feedback given by the apps may encourage users to continue and reinforce their engagement.

Moreover, the effect of the “law of attrition” can be a confounding factor in eHealth studies [18]. Yet, thorough analysis of these factors can explain the difference between the optimism of the designers and the reality of the rate of use. These considerations should drive the development of a digital tool as well as the interpretation of study results.

Our main objectives were to evaluate the usefulness, usability, and reliability of our smartphone app during a 6-month period (level 4 of the American Psychiatric Association hierarchical framework) to determine the acceptability of the app and to qualitatively evaluate the factors that could explain differences in use (in accordance with the principles of specific attrition bias [18]).

## Methods

### Ethics

Ethical approval was obtained from the *Comité de Protection des Personnes, Ouest V* (January 2017). All participants gave consent.

### Participants

Parents of individuals diagnosed with autism spectrum disorders, using Autism Diagnostic Interview-Revised diagnoses based on National Health Authority recommendations [19], from 3 to 16 years of age (at the time of the study) in the *Pays de la Loire* region of France were recruited. All families at the Department of Child and Adolescent Psychiatry of *Centre Hospitalier Universitaire* de Nantes and from the Regional Center for Autism (*Pays de la Loire*) were eligible. Inclusion criteria were having a child with an autism spectrum disorder diagnosis based on International Classification of Diseases tenth revision criteria, having a smartphone (iOS Apple or Android), and signing the consent form for participation. Exclusion criteria were having several children with autism spectrum disorder diagnoses, having children living in more than 2 houses, having an old smartphone with which there would be a significant decrease in user experience, or having no personal smartphone. We included the first 100 families that agreed to participate and that met the criteria. Because the study was exploratory, we decide that it was not necessary to randomize patients.

### Data Security

To obtain approval from the National Center for Informatics and Liberty, we designed a secure data-handling pathway. Data were stored in the app on parents’ smartphones and were inaccessible by unauthorized people (an individual code was required when the app was opened). Families included in this study transmitted these data to the investigators using a strict, secure 5-step process: (1) Data in the smartphone of the participant (in-app coding) were encrypted with a 16-digit encryption key and a personal temporary code for access to data. (2) Encrypted data were transmitted to a secure server (Ivory Healthcare Inc). (3) Data were physically transferred by USB stick or disk from the server to Nantes University Hospital secure medical server at the end of the study period. (4) Once the data were uploaded on the intranet of the University Hospital, decryption was performed *in situ* using specific software after the principal investigator entered a short automatically generated validation code. (5) Data were organized in a data spreadsheet for analysis and calculation.

### App

The idea for the Smartautism [17] app emerged during discussions with associations of parents of people with autism, who were informally involved in the development process. The acceptability assessment of Smartautism was one step of a large digital empowerment project. The app is a combination of ecological momentary assessment with feedback that may be used by parents to adapt their educational behavior. The parents must provide regular ratings (mandatory twice a week but additional on-demand ratings were possible if needed by the parents) for a long period, and the feedback screen provides a synthesis of the ratings.

**Figure 1:**
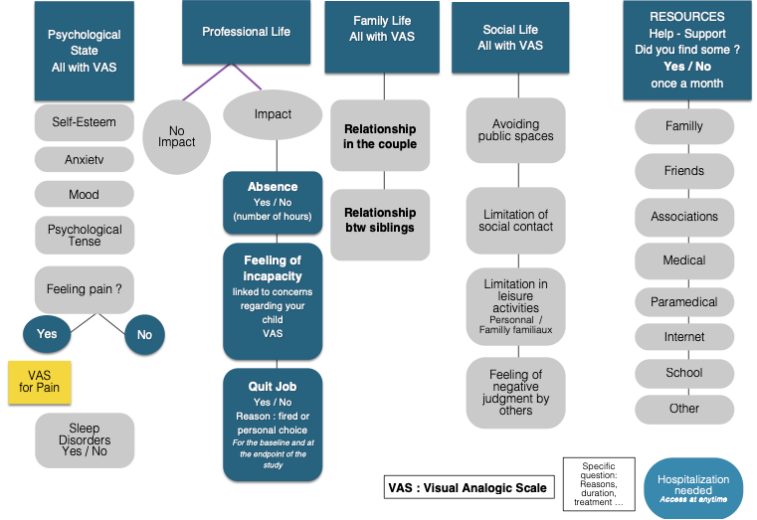
Smartautism app questions for parents (1b) about their children and themselves (1a) (image adapted from [17]).

**Figure 2:**
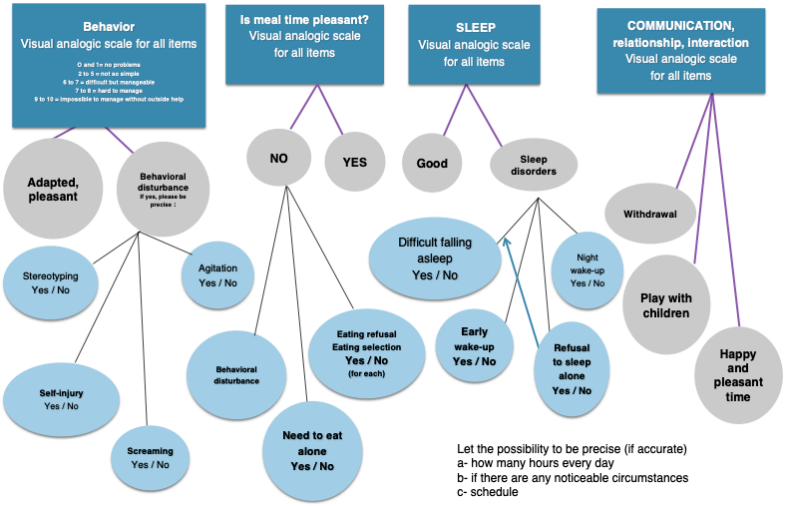
Smartautism app questions for parents about their children, two times every week (image adapted from [17]).

Parents rated behavior in several day-to-day basic situations (meal, lunch, etc) and answered questions (Figures 1 and 2) in the app. Parents had the opportunity to answer ecological questions about their children and their own psychological state [17]. The feedback screen converted user scores to graphs in order to allow users to data visualize their responses to provide an overview of the information.

Smartautism is freely downloadable from the Apple Appstore and the Google Play Store but requires a key code for access.

### Study Design

We conducted a prospective longitudinal exploratory open study. We collected qualitative and quantitative data over a 6-month period to assess acceptability of the app. Acceptability has several aspects. In digital technology, it may be reduced to 4 dimensions: (1) usefulness, (2) usability, (3) reliability, and (4) risk [10]. Usefulness reflects intent to use, which is a good predictor of usage behavior. Acceptability encompasses several concepts, for example, in ISO standards [20], usability comprises 3 dimensions: effectiveness, efficiency, and satisfaction.

To assess usefulness and intent to use, our primary outcome was overall response rate, *R* = (*number of dates with responses)* / (*possible instances with responses over the 6-month period)*. In this case, there were 60 possible instances. All data were directly extracted from the app.

### Primary Analysis

Completion rate was categorized into 5 levels: 100%, 99% to 80%, 79% to 50%, 49% to 20%, and <20%. The choice of intervals was not based on a specific theory, but on facilitating ranking of user behavior; the size of the intervals was modeled on Cohen effect size intervals.

We were able to determine the total number of views of the feedback screen because the screen requires activation by the user. Raw attrition proportions at different steps in time can be illustrated as attrition curves, and the shape of these curves (logarithmic or sigmoid) allows formulation of hypotheses about the causes of attrition [18].

We assessed attrition criteria [18] in the form of a questionnaire (Table 1). This questionnaire was given to users at the end of the 6-month period. Responses were rated on a 5-point Likert scale (where 1 indicated the worst satisfaction and 5 indicated high satisfaction).

We categorized patients according to their completion rate (high: ≥80%, moderate: 20-80, and low: <20) and used the Kruskal-Wallis test to isolate questions for which there was at least1 difference between groups (high, moderate, low). To determine posthoc stochastic dominance, we used the Dwass-Steel-Critchlow-Fligner pairwise comparison test.

**Table 1:** Use and user experience question content (adapted from [18]).

Item	Question content	Impact on nonusage and dropout attrition rate
1	Quality and relevance of information given before the trial	If low, risk of unrealistic expectations which results in a disengagement
2	Ease of the inclusion process (consent, implementation)	Quality of recruitment affects attrition. if it is too easy to enroll then the dropout rate may be high
3	Ease of drop out/stop using it	This parameter can negatively influence the use of the app
4	Ease of use and reliability of the technical interface	Poor usability (complexity of the interaction between an object and its user) contributes to a high rate of attrition
5	“Incentive” or “push” factors (callbacks, reminders, research assistants chasing participants)	This parameter can positively influence the use of the app (staying more in the trial)
6	Personal contact (during registration and inclusion) via face-to-face or by phone, rather than virtual contacts	Human contact promotes the use of the app
7	General quality of the feedback information and of the information summary screen	Positive feedback and encouragement positively influence the use of the app
8	Perceived benefits of interest in completing the study	Motivational factor that decreases attrition
9	Free to use	Paying more commits the user and decreases attrition
10	Time and workload required by the apps	If the burden is too high, it may result in higher attrition
11	Existence of concurrent interventions (web, therapy)	Risk that the user no longer perceives the specific interest of the app
12	Major life events, or of society, which could have stopped using the app	Lead to distraction and nonuse by shifting priorities
13	Experience of the other user (or being able to obtain help)	Indirectly through to dropout and nonusage

### Secondary Analysis

Based on initial results that showed different app use behaviors, we separated participants into 2 groups. Group A comprised participants who consistently had completion rates above 50%, and group B comprised participants who consistently had completion rates below 50%.

## Results

### General

**Figure 3:**
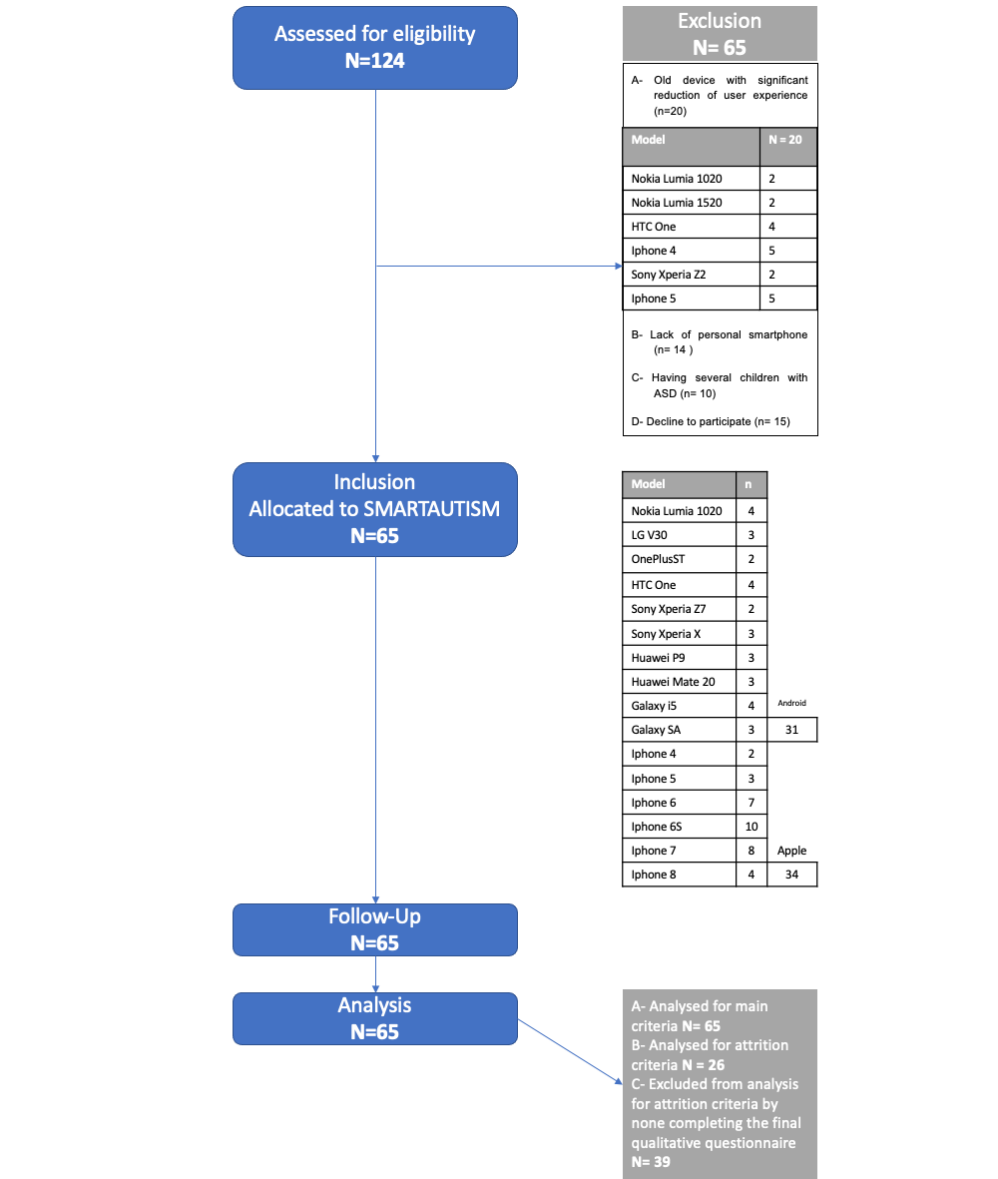
Flow diagram.

A total of 124 families were consecutively screened during an 18-month recruitment period (Figure 3), of which, 65 families, with 46 boys and 19 girls, were included in our study (Table 2).

The overall response rate was high (3698/3900, 94.8%). Of the 3900 instances (for n=65 participants), only 1347 were completed in full, while 837 instances were more than 80% complete, 509 instances were between 50% and 79% complete, 897 instances were between 20% and 49% complete, and 310 instances were less than 20% complete (of which, 202 were 0% complete).

**Figure 4:**
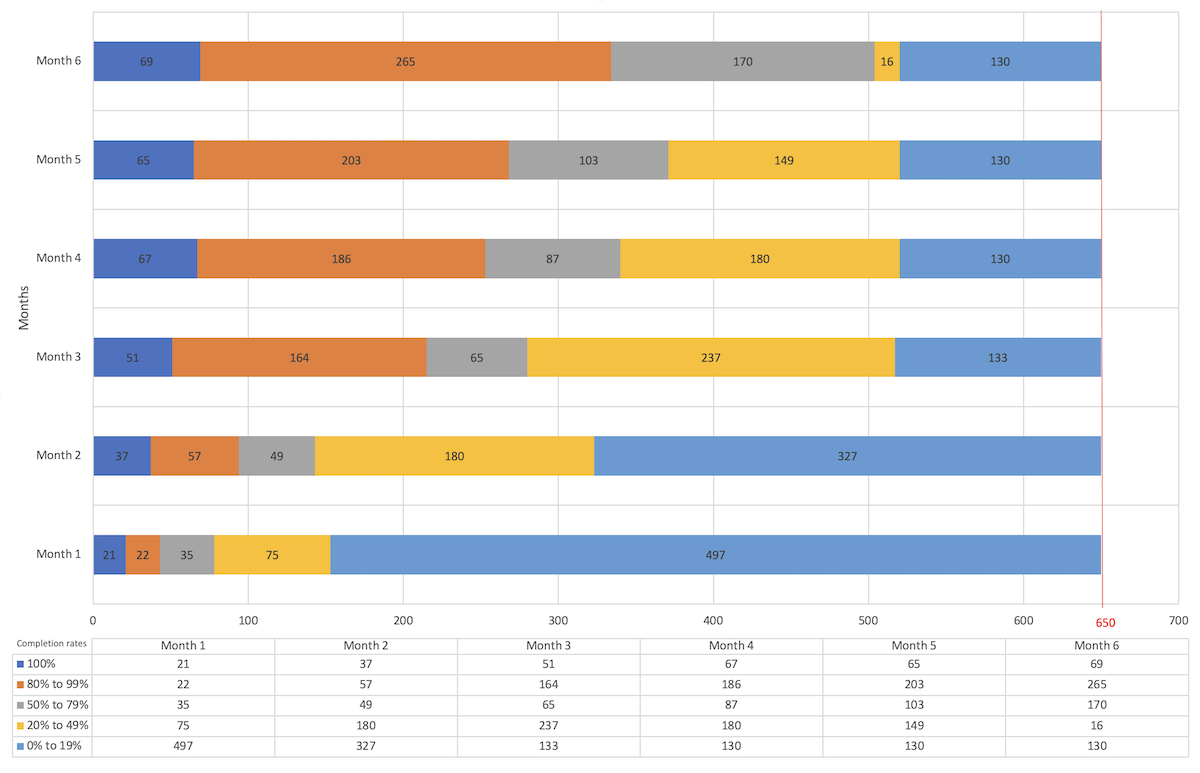
Distribution of the answers by month of use and completion rate (each month 650 answers were expected).

The number of responses completed by participants tended to decrease over time, mainly after the third month (Figure 4). However, responses were consistently completed throughout the study by participants who completed over 90%. Only 13 of the 65 participants (20%) completed all responses. Of the 65 participants, only 1 participant had a 0% completion rate, while 17 participants (26%) had completion rates that remained above 50%, and 34 participants (52%) had progressively decreasing completion rates. Overall, 46% of participants (30/65) consistently had completion rates over 50%, and 54% (35/65) had completion rates under 50%.

**Table 2:** Study population of parents with children with autism spectrum disorders.

Characteristics		Value (n=65)
Age at diagnosis^a^ (months), mean (SD)		20.3 (6.3)
Age of the father (years), mean (SD)		34.52 (6.52)
Age of the mother (years), mean (SD)		33.63 (3.32)
Age of the children (years), mean (SD)		7.56 (4.52)
**Associated disease^b^, n (%)**		
	None	52(80)
	Epilepsy	8 (12.3)
	Chromosomal abnormalities	9 (13.8)
	Endocrine	2 (3)
**Gender of the children, n (%)**		
	Male	46 (72)
	Female	19 (28)
**Phone, n (%)**		
	Apple iPhone	34 (52.3)
	Android	31 (47.7)

^a^Autism Diagnostic Interview-Revised assessed diagnosis.

^b^Some patients may have >1 association; therefore, percentages do not add to 100%.

### Grouped by Usage

The distribution of Android and Apple smartphones for participants with completion rates >50% (group A; Android: 48%, Apple: 52%) was similar to that for participants with completion rates <50% (group B; Android: 46%, Apple: 54%).

The ages of the fathers and mothers in group A (father: mean 24.3 years, SD 3.5; mother: mean 25.5 years, SD 4.0; *P*=.01) were lower than those in group B (father: mean 29.0 years, SD 6.0; mother: mean 32.0 years, SD 6.4; *P*=.03). We did not find any significant differences for age of the child (*P*=.31) or city type (more or less than 20,000 inhabitants: *P*=.117).

**Figure 5:**
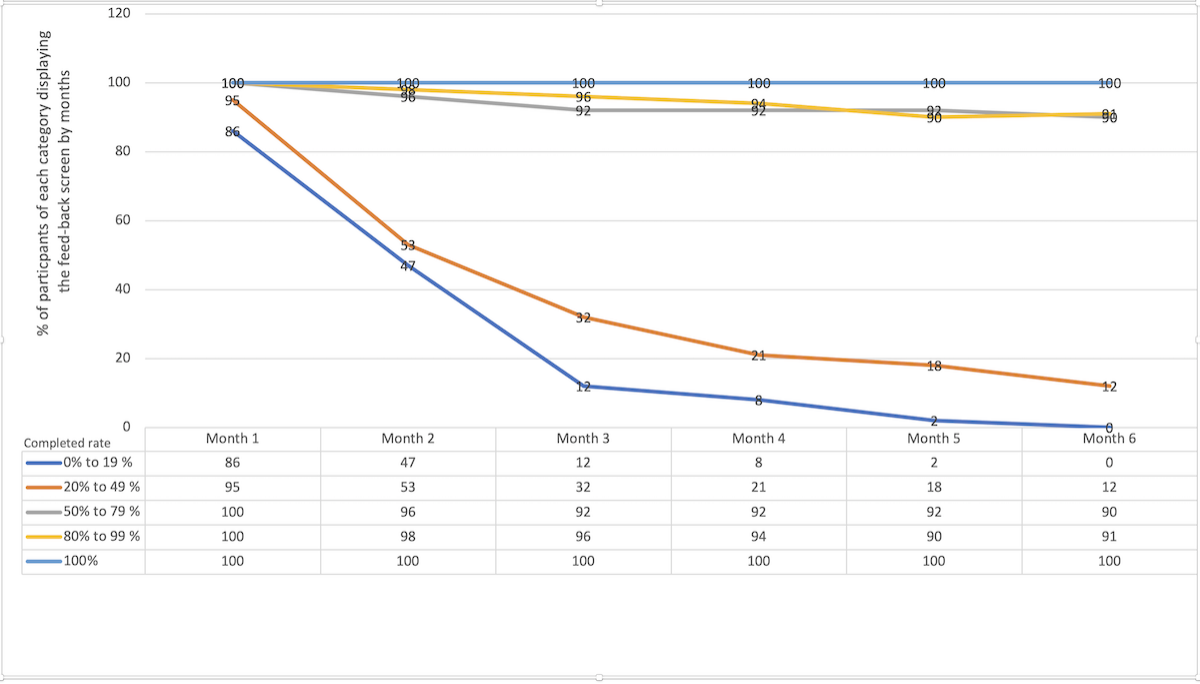
Participants who displayed the feedback screen by month, based on their completion rate.

The individuals most likely to fully answer the questions were those who were most likely to display the feedback screen (Figure 5). There were 39 attrition questionnaires with responses: 9 participants were low users, 3 participants were moderate users, and 27 participants were high users.

There were 7 questions (questions 1, 2, 4, 5, 7, 8, and 10) with between-group differences (Table 3). For question 7, which evaluated user satisfaction with the feedback screen, there were significant differences both between high and moderate (*P*=.007) and high and low (*P*<.001) users; however, the difference between low and moderate users was not significant (*P*=.14).

**Table 3:** Comparison between high, moderate, and low users of the Smartautism app for each item of the attrition questionnaire.

Item				Pairwise^a^				Between-group				
				*W* test statistic		*P* value		Chi-square (*df*)		*P* value		Effect size
**1**								19.70 (2)		<.001		0.519
	High	Moderate	−3.88		.02							
	High	Low	−5.72		<.001							
	Moderate	Low	—^b^		—							
**2**								19.70 (2)		<.001		0.519
	High	Moderate	−3.88		.02							
	High	Low	−5.72		<.001							
	Moderate	Low	—		—							
3								—		—		—
**4**								30.10 (2)		<.001		0.792
	High	Moderate	−4.92		.001							
	High	Low	−7.10		<.001							
	Moderate	Low	4.69		.003							
**5**								8.76 (2)		.01		0.231
	High	Moderate	−1.27		.64							
	High	Low	−3.99		.01							
	Moderate	Low	−1.56		.51							
6								—		—		—
**7**								26.45 (2)		<.001		0.696
	High	Moderate	−4.27		.007							
	High	Low	−6.57		<.001							
	Moderate	Low	−2.71		.135							
**8**								32.31 (2)		<.001		0.850
	High	Moderate	−5.68		<.001							
	High	Low	−7.51		<.001							
	Moderate	Low	−1.56		.51							
9								—		—		—
**10**								29.06 (2)		< .001		0.765
	High	Moderate	−4.92		.001							
	High	Low	−7.04		<.001							
	Moderate	Low	0		>.999							
11								—		—		—
12								—		—		—
13								—		—		—

^a^Pairwise comparisons are presented for significant items.

^b^Missing or unquantifiable data.

## Discussion

As expected from previous literature [21], in our study population, there were more male children than female children with autism spectrum disorder. We found that participants had a very high intent to use the app during the 6-month period (3698/3900, 94.8%); however, secondary analysis showed that only 46% of participants (30/65) consistently had completion rates over 50%. High users were characterized by having higher satisfaction (question 7) with the feedback screen when compared to low (*P*<.001) and moderate (*P*=.007) users.

These results are consistent with those in previous studies [13] on real-life use of smartphone apps in digital medicine and in psychiatry, which show disappointing use rates despite the high expectations of professionals. We did not take the high overall response rate (94.8%) into account in our interpretations and discussions because we recruited families and patients who were already very dedicated to our facility. There were many screened families (59/124 47.5%) that were not included in the study because they lacked a smartphone or had old devices, despite the fact that overall rate of 79% of people in France have smartphones [22]. Moreover, the distribution of smartphone type in our study population was not representative of that of the French population—half of the participants used iPhones, whereas iPhones are used by only 15% of the population in France [22].

Despite encouraging results, our study shows that half of the users (35/65, 54%) did not use the empowerment app regularly. We can assume that the specific parent population in our study (known from our facility) was intrinsically motivated; therefore, we can consider several reasons to explain the constant gap between real usage and expectation in digital health care [23]: (1) the app did not offer sufficient gain for the families for regular use, which is suggested by the fact that only high users were the most interested by the feedback screen, (2) security concerns are always present even if we did explain (orally and with notice inside the app) the data protection measures that we used, and (3) design and ergonomics were tidy and elegant in our app, but we suspect it is very difficult to develop an app that can compete with those developed by large corporations and used in everyday life by our population.

Often, individuals use health apps only for a short period of time. This “law of attrition,” corresponding to the loss of participants during an experiment, raises some questions [18]. Eysenbach [18] highlighted that this rate decreases if a user perceives a relative advantage with the app (perception that this innovation is superior to the idea that it replaces). Our attrition questionnaire results (responses from partial study population: 39/65, 60%) also strongly suggest that reminders and feedback, when appreciated and accepted, are a strong motivation and may drive participants to be high users instead of low users. If engagement can be viewed as a product of experience or interaction, the presence of push factors (reminders) or positive feedback appears to be efficient for engagement.

Recently, the components of engagement in technology, in particular with respect to apps, were evaluated and a user scale based on 4 dimensions was proposed [24]: focused attention, perceived usability, aesthetic appeal, and reward. The *reward* dimension corresponds to the hedonistic aspects of the experience; the feedback screen could enhance this dimension of the app when used; therefore, this component needs to be further developed.

Low users demonstrated low levels of satisfaction in various areas. They expressed reluctance to spend too much time using the app (question 10, high vs low: *P*<.001). They also were annoyed by technical issues (question 4, high vs low: *P*<.001). It appears that too much complexity in the interaction between an app and its users may contribute to a high rate of attrition. These parameters can negatively influence the use of the app. Interestingly, low users were also significantly more dissatisfied by the information and inclusion process of the study.

Perfect engagement with apps will never exist; O’Brien and Toms [25] argue that engagement is not a static, but multistage, process—with a point of engagement, then a period of engagement, a point of disengagement, and a period of reengagement. Moreover, when behavioral symptoms are not present, engagement may decrease due to lack of necessity. Our results show that motivation for long-term use is strongly associated with perceived benefits in completing the study (high vs moderate: *P*<.001; high vs low: *P*<.001), which emphasizes the importance of precise framing during the patient inclusion process (quality of information, explanation of consent, purpose, and benefit). There is a need for stronger collaboration between academics and digital specialists to provide accurate empowerment tools. Indeed, laws of attrition encompass classic motivational rules for study enrollment and new digital constraints related to technical aspects (design, acceptability, stability) [18]. App development requires academic studies that take laws of attrition into account, mainly because, for the past decade, empowerment and enhancement of patients’ responsibilities in their own health care have been growing [26]. Our app is only a first step, but it is consistent with the on-going shift toward the use of such technologies in psychiatry, especially for outpatients.

The Smartautism app is a first and encouraging step in digital empowerment for families of individual with autism spectrum disorders. Our results suggest that users need to perceive the utility of digital tools in order to use them. We plan to add an advice section, through the feedback screen, providing guidance and suggestions generated by Bayesian network algorithm [27] because more powerful algorithms (enhanced by artificial intelligence) might be useful in providing accurate and personalized advice for users.
